# Factors Associated with Gaps in Naloxone Knowledge: Evidence from a 2022 Great Plains Survey

**DOI:** 10.21203/rs.3.rs-3536993/v1

**Published:** 2023-11-03

**Authors:** Spencer Cooper-Ohm, Patrick Habecker, Ryan Humeniuk, Rick A. Bevins

**Affiliations:** University of Iowa; University of Nebraska-Lincoln; Ohio University; University of Nebraska-Lincoln

**Keywords:** Naloxone, Narcan^®^, Cascade of Care, United States, Great Plains, Harm Reduction, Stigma

## Abstract

**Background:**

The rising prevalence of fast-acting opioids in the United States suggests the increased need for non-first responder administration of naloxone. Effective administration of naloxone during an overdose requires that bystanders are familiar with, have access to, and know how to use naloxone.

**Methods:**

Drawing on the 2022 Nebraska Annual Social Indicators survey, we analyzed naloxone familiarity, access, and competency to administer among a statewide, address-based sample of Nebraskan adults.

**Results:**

There were significant gaps in naloxone knowledge in Nebraska. Although 75.6% of respondents were familiar with naloxone, only 18.6% knew how to access naloxone and 17.6% knew how to use naloxone. We find that more frequent religious service attendance is associated with lower odds of naloxone familiarity. Among those familiar with naloxone, a higher perception of community stigma towards opioids generally is associated with lower odds of naloxone access and competency. Higher perception of community stigma towards heroin, methamphetamines, and cocaine, however, is associated with higher odds of naloxone access. Finally, past overdose experience, lifetime illicit opioid use, being close to a person who uses opioids, and having access to illicit opioids was not significantly associated with naloxone familiarity, access, or competency among respondents in Nebraska’s two largest cities, Omaha and Lincoln. Outside of these cities, past overdose experience and access to illicit opioids was associated with higher odds of naloxone access and competency, but lifetime opioid use and being close to a person who uses opioids had no effect.

**Conclusions:**

Our findings highlight the continued need for education on naloxone with a specific focus on access and competency to further reduce opioid-related overdose deaths. Education campaigns targeted at places of worship or individuals close to people who use opioids may further serve those with a lower likelihood of naloxone familiarity and promote knowledge of naloxone among those with higher odds of encountering an overdose. Further work is needed to understand differences in the relationship between substance-specific perceived stigma and its association with naloxone access.

## Background

More than 80,000 people died due to opioid-involved drug overdoses in 2021, reflecting a 15% increase from 2020 (CDC, 2022), which was itself an increase of 30% from 2019 (Hedegaard et al., 2021). These recent increases are a continuation of trends in deaths associated with synthetic opioids that began in 2014. This corresponds with a similar rise in the availability of illicitly manufactured fentanyl: a synthetic opioid roughly 100 times as potent as morphine (Rudd et al., 2016; Volpe et al., 2011). Despite the nationwide growth in fentanyl-related overdoses, opioid deaths in Nebraska have remained relatively low. In 2021, Nebraska had an opioid-related overdose death rate of 5.8 per 100,000, compared to the national average of 24.2 and Midwestern average of 24.9. Despite a lower death rate, fentanyl-involved deaths comprised a majority of overdose deaths in Nebraska (79.2%), similar to Midwest (90.8%) and national estimates (88.0%) (CDC, 2023). Amid such major growth in the national fentanyl supply and overdoses, Nebraska’s still small opioid overdose death rate suggests that Nebraska may have an especially opioid naïve population compared to the rest of the United States.

Rapid access to naloxone, an opioid antagonist medication that can reverse the effects of an ongoing overdose, is critical, especially in situations involving high potency opioids. Previously, naloxone distribution was primarily focused on first responders, medical professionals, and other professions that had high contact with overdose situations. However, as fentanyl overdoses can occur quickly—sometimes within two minutes and among people that may be unaware they ingested fentanyl—the distribution of naloxone to the general population has become more important (Fairbairn et al., 2017; McKnight et al., 2023). A recent meta-analysis study found that median emergency medical services response time in the United States is nine minutes (Alruwaili & Alanazy, 2022) and often longer in rural areas (Cabral et al., 2018). Non-professional administration of naloxone at the scene of an overdose can bypass potentially deadly emergency response delays. Because the proportion of opioid overdoses requiring two or more doses of naloxone to reverse has been increasing since 2013 (Abdelal et al., 2022; Schneider et al., 2021; Somerville et al., 2017) increasing both the proportion of people carrying naloxone and the number of doses they carry is critical to reducing opioid overdose deaths.

In response to rising deaths, states have been rapidly increasing access to naloxone beyond first responders and medical professionals. Between 2013 and 2017, all fifty states and Washington D.C. enacted laws that allow dispensing naloxone either through pharmacist prescribing, standing order, or direct legislative authority (Prescription Drug Abuse Policy System, 2022). These laws corresponded with skyrocketing rates of pharmacy dispensing of naloxone, from a national average of 0.55 naloxone prescriptions per 100,000 in 2012 to 292.31 in 2019. Nebraska naloxone prescription rates have remained relatively low compared with national rates, with 95.39 prescriptions per 100,000 in 2019 (Guy et al., 2021). On a national scale, the Food and Drug Association approved Narcan and RiVive, two naloxone nasal sprays, for over-the-counter sale in 2023 (FDA, 2023a; FDA, 2023b).

Attaining a high level of general population naloxone readiness requires a series of knowledge, access, and training goals. In short, people need to know naloxone exists, have access to naloxone, be trained to use naloxone, have used naloxone, and carry naloxone frequently. These steps make up the “naloxone treatment cascade” (Tobin et al., 2018). Despite the increased nationwide distribution of naloxone, there are currently significant gaps in the naloxone treatment cascade. Studies find that although most surveyed adult Americans are familiar with naloxone, only a small proportion are aware that naloxone can be obtained in pharmacies (Hohmann et al., 2022; Schneider, 2021; Tobin, 2018). Gaps in naloxone coverage also exist among people who use drugs (PWUD): a 2021 meta-analysis of studies in areas across North America and Europe found that although 57% of PWUD owned naloxone, only 20–28% carried it on a regular basis (Burton et al., 2021). In this study, we focus on the first three steps of the naloxone treatment cascade: naloxone familiarity, access, and competency to administer (referred to collectively as “naloxone knowledge”).

Several studies have analyzed factors that influence naloxone knowledge and possession among PWUD, typically through recruiting participants in treatment programs, Syringe Service Programs (SSPs), or detoxification units (Gicquelais et al., 2019; Jones et al., 2021; Kirane et al., 2016; Tobin, 2018) or respondent-driven sampling (Lipira et al., 2021; Reed et al., 2019). Studies have found that participants with prior overdose or naloxone administration experience tend to have more knowledge about naloxone (Gicquelais, 2019; Kirane, 2016; Tobin, 2018) and that female participants are more likely to carry naloxone on a regular basis (Lipira, 2021; Tobin, 2018).

Research on familiarity with naloxone among people who do not regularly use drugs is limited and fails to directly measure a key drop off in the naloxone treatment cascade: knowledge of where to access naloxone. Two studies, an online panel survey and a survey distributed in the Richmond, VA area, measured naloxone familiarity and knowledge that naloxone is sold at pharmacies, but not if participants knew a source for naloxone (Haggerty et al., 2018; Hohmann, 2022). This distinction is especially important because knowledge that pharmacies sell naloxone does not necessarily lead to an individual being able to find naloxone. Several studies have found that factors like an area’s wealth and rurality correlate with ‘naloxone deserts’: pharmacies that have authority to dispense naloxone but do not keep it in stock (Abbas et al., 2021; Green et al., 2017; Lozo et al., 2019).

A 2020 study measuring covariates of naloxone prescription in Ontario is closer to measuring the outcome of naloxone access, but Canada’s free naloxone policy and social differences between the two countries make it difficult to directly apply the study’s results to the United States (Antoniou et al., 2020). Finally, two studies have analyzed public opinion on naloxone, but these studies either assumed participant knowledge of naloxone or provided participants with information about naloxone before the survey (Agley et al., 2022; Calabrese & Bell, 2019). These studies, although insightful into public perception of recent harm reduction policy, do not provide information on naloxone knowledge.

Our study aims to expand on the literature surrounding naloxone awareness with a specific focus on the state of Nebraska’s unique place in the opioid epidemic by measuring Nebraskans’ naloxone familiarity, access, and competency. Nebraska expanded public access to naloxone in May 2015 (Nebraska LB390, 2015). It currently has 112 pharmacies participating in the state’s free naloxone program and a free online service that ships Narcan to Nebraska addresses upon request. (Stop Overdose Nebraska, 2023). Despite this progress, Nebraska’s network of naloxone distribution is still lacking in several respects. In 2022, Nebraska was one of six states without any SSPs and in 2019 had the lowest per-capita pharmacy naloxone dispensation rate of all 50 states (Guy, 2021; Legislative Analysis and Public Policy Association, 2022). Our study examines naloxone knowledge in an area lacking several standard naloxone distribution methods and to our knowledge is among the first to examine statewide variation in factors that influence naloxone knowledge in both people that do and do not use drugs. A prior study measured factors influencing naloxone access based on a 2020 survey of Nebraskans (Schlosser et al., 2022). We built on this research by using more recent data, adding measures of naloxone familiarity and competency to our analysis, and accounting for new variables in our model including the respondent’s perception of community stigma towards PWUD.

## Methods

Data for this project comes from the Nebraska Annual Social Indicators Survey, an omnibus mail survey sent to an address-based sample of 8,000 Nebraskan adults. In 2022, the sample frame was stratified into 8 regions with 1,000 addresses per region. The 8 strata are based on the 6 behavioral health regions of the state, and a further 2 that separately capture the two largest cities in the state, Omaha and Lincoln. The adult in the household with the next birthday after July 1, 2022 was asked to complete the survey either online or by returning the survey packet. Data was collected between July and November 2022. Full sampling methodology and survey instruments are available through the University of Nebraska-Lincoln Bureau of Sociological Research (Bureau of Sociological Research, 2021).

A total of 1,455 completed or partially completed surveys were returned, for an AAPOR Response Rate 2 of 18.2% (AAPOR, 2023). To account for the stratified sample design, data were weighed by stratum, within-household probability of selection, and non-response rate. Post-stratification weights were assigned based on region, age, and sex. 50.9% of survey responses had at least one missing value on measures in this paper, excluding forced skips. The variables with the highest levels of a missing item were annual income (9.97% missing), political orientation (9.69%), religious affiliation (7.35%), and access to heroin (6.8%). To avoid listwise deletion, missing values were estimated with 50 chained multiple imputations with the mi command suite in Stata 17 and a seed of 68588.

Our primary dependent variables come from two questions on the survey. *“Do you know where to get Narcan (naloxone) if you needed it?”* (Yes/No/I don’t know what this is) and *“Do you know how to use Narcan (naloxone)?”* (Yes/No). Respondents were instructed to skip the second question if they answered “I don’t know what this is” to the first question. Respondents were categorized as having familiarity with naloxone if they answered “Yes” or “No” to the first question, and without familiarity if they answered “I don’t know what this is.” Naloxone access was determined by participants answering “Yes” to the first question. Finally, respondents were categorized as having naloxone competency if they responded “Yes” to the second question, and without competency if they answered “No” to the second question or responded “I don’t know what this is” to the first question and skipped the second question as instructed.

Perception of community stigma towards drug use was measured using an adaptation of the “awareness” portion of the brief opioid stigma scale (Yang et al., 2019). Our scale used the average value of four questions on a 5-point Likert scale: “Strongly disagree,” “Disagree,” “Neither disagree nor agree,” “Agree,” and “Strongly agree” with a value of 1 corresponding to “Strongly disagree” and 5 to “Strongly agree.” Respondents rated their agreement with the assertions that people in their community believe that a person who uses opioids “cannot be trusted,” is “dangerous,” “to blame for their own problems,” and “lazy.” The same questions were asked regarding a person who uses “cocaine, methamphetamines, and heroin.” While these categories overlap (heroin is an opioid), assessing community stigma in this fashion allowed respondents to differentiate between stigma towards opioids at large and stigma towards explicitly prohibited drugs. Other variables included yes/no answers to having used illicit opioids or heroin in their lifetime, being close with someone that currently used illicit opioids or heroin, knowing someone that experienced an overdose in the past year, having access to illicit opioids or heroin, and knowing what SSPs are.

The survey also collected information on age, sex, race, ethnicity, highest education obtained, household income, partner status, number of children present in the household, political orientation, religious affiliation, residence (Farm/Open country, but not a farm/Town or city) and rurality based on Core-Based Statistical Areas (Metropolitan/Micropolitan/Outside). Due to sample size restrictions, we divide race and ethnicity into White non-Hispanic and non-White/Hispanic categories. Household income is divided from $0-$30,000, $30,001-$100,000, and $100,001+. This roughly corresponds to the bottom, middle two, and top quartiles of income returns in Nebraska (Nebraska Department of Revenue, 2020). Education is divided into groups with no college education, some college/technical degree/associate’s degree, and a bachelor’s degree or higher. Respondents were asked how often they attended religious services with eight response options, ranging from “Several times a week” to “Never.” We reverse coded responses so 7 represents the highest attendance frequency and treated the resulting recode as a continuous variable.

We used logistic regression to predict likelihood of naloxone familiarity. Then, restricted to respondents with naloxone familiarity, we predicted respondents’ likelihood of naloxone access and naloxone competency. Then, we performed the same analysis for respondents located in Omaha and Lincoln (Nebraska’s two largest cities) and separately for respondents in all other regions. Analyses were conducted in Stata 17 with the *svy* command for sample design and weights, and mi commands for multiple imputation.

## Results

Table 1 shows descriptive statistics of our sample. The majority of respondents reported knowing what naloxone is (75.60%) but few knew where they could access naloxone (18.59%) or how to use it (17.61%). Among those with naloxone familiarity, 24.46% of respondents knew where to access naloxone and 23.02% knew how to use it. Few respondents knew someone who experienced an overdose in the past year (5.87%), were close to a person who uses illicit opioids or heroin (6.30%), or used illicit opioids or heroin in their lifetime (6.56%). 30.84% of respondents were familiar with SSPs.

After survey weights, our sample had an above average proportion of White and educated respondents. Non-Hispanic White respondents made up 90.81% of our sample compared to 76.9% of the Nebraska population at large, and survey respondents with a bachelor’s degree or higher made up 53.84% of our sample compared to 39.2% in Nebraska (U.S. Census Bureau, 2022). Our sample was majority employed (75.8%), married or with a partner (75.18%), lived in a town (83.14%), and in a metropolitan area (68.4%).

Results from our logistic regression can be found in Table 2. We found that familiarity with SSPs was associated with higher odds of familiarity with naloxone (OR = 1.809, p = .015). Having a technical degree or some college compared to no college education (OR = 1.748, p = .027), and an annual income above $100,001 compared to an income below $30,001 (OR = 2.112, p = .032) was associated with higher odds of naloxone familiarity. In contrast, we found no significant difference in odds between having a bachelors or terminal degree compared to having no college, or an annual income between $30,001-$100,000 compared to an income less than $30,001. More frequent religious attendance was associated with lower odds of naloxone familiarity (OR = .875, p = .004).

Restricting our analysis to respondents familiar with naloxone, analysis shows that familiarity with SSPs (OR = 2.649, p < .001), being employed compared to unemployed (OR = 1.957, p = .016), and having access to illicit opioids or heroin compared to not having access (OR = 2.274, p = .006) was associated with a higher likelihood of naloxone access. Higher perceived stigma towards people who use opioids was associated with lower odds of naloxone access (OR = 0.464, p = .001), while higher perceived community stigma towards heroin, methamphetamines, and cocaine was associated with higher odds of naloxone access (OR = 2.154, p = .002).

Among those familiar with naloxone, familiarity with SSPs (OR = 3.997, p < .001) and being Catholic compared to Protestant (OR = 1.921, p = .039) was associated with higher odds of naloxone competency. Higher perceived community stigma towards people who use opioids was associated with lower odds of naloxone competency (OR = 0.611, p = .036).

We then used subpopulation analyses to replicate models from Table 2 while restricting the sample into two groups: Omaha and Lincoln combined, and the remainder of the state. Our variables measuring respondents’ Core-Based Statistical Area and residence were dropped due to collinearity. In regions outside of Omaha and Lincoln (Table 3b), knowing someone that experienced an overdose in the past year (OR = 4.806, p = .001) and having access to illicit opioids or heroin (OR = 3.234, p = .001) was associated with higher odds of naloxone access among those familiar with naloxone. Among those familiar with naloxone, being older (OR = 1.022, p = .044) and having an annual income of more than $100,000 compared to less than $30,000 (OR = 2.799, p = .021) was associated with higher odds of naloxone access, but income above $100,000 was not positively associated with naloxone familiarity as it was in our statewide model. Knowing someone that experienced an overdose in the past year (OR = 3.465, p = .009) and having access to illicit opioids or heroin (OR = 2.166, p = .036) was associated with higher odds of naloxone competency. Perceived community stigma towards people who use opioids or heroin, methamphetamine, and cocaine was not significantly associated with odds of naloxone familiarity, access, or competency.

In Omaha and Lincoln (Table 3a), being employed compared to unemployed was associated with lower odds of naloxone familiarity (OR = 0.326, p = .016), but among those with naloxone familiarity, being employed compared to unemployed was still associated with higher odds of naloxone access (OR = 4.505, p = .006) as it was in our statewide model. Among those familiar with naloxone in Omaha and Lincoln, higher perceived community stigma towards people who use opioids was associated with lower odds of naloxone access (OR = 0.371, p = .009) while higher perceived stigma towards people who use heroin, methamphetamines, or cocaine was associated with higher odds of naloxone access (OR = 3.252, p = .009). In Omaha and Lincoln, knowing someone who experienced an overdose in the past year, lifetime illicit opioid or heroin use, being close to someone who uses illicit opioids or heroin, and having access to illicit opioids or heroin was not significantly associated with odds of having naloxone familiarity, access, or competency.

## Discussion

Our findings corroborate prior studies that reported large gaps in the naloxone treatment cascade between familiarity and possession of naloxone. Although 75.6% of respondents in our sample of Nebraskan addresses knew what naloxone is, only 18.6% of the total sample knew where they could access it and 17.6% knew how to use it. Among respondents who were familiar with naloxone, 24.46% knew where to access it and 23.02% knew how to use it. Naloxone familiarity in Nebraska is nowhere near universal, and rates of naloxone access and competency within the state still have significant room to grow.

Higher perceived community stigma towards people who use opioids is significantly associated with a decrease in odds of naloxone access and competency. Prior qualitative surveys of pharmacists found that stigma is perceived as a barrier to naloxone access (Bounthavong et al., 2020; Gatewood et al., 2016; Green, 2017; Spivey et al., 2020). A survey of people who inject drugs found similar results: perceived stigma towards opioids and people who use opioids discourages people who use opioids from seeking syringes and naloxone, especially in pharmacies (Paquette et al., 2018). We found that this stigma effect may extend to people who do not use drugs as well. Community stigma towards people who use heroin, methamphetamines, and cocaine has the opposite effect: higher perception of stigma leads to a higher likelihood of naloxone access. To our knowledge, this effect has not been observed in the literature before and is especially notable given its contrast with the effect of higher community opioid stigma.

Our results are likely partially capturing differences in stigma by substance type. Heroin, methamphetamines, and cocaine are explicitly illegal, while referring to the category of ‘opioids’ more generally includes legally obtainable prescription opioids. These drug types also contain drastically different cultural connotations: prescription opioids are pharmaceutical in nature and are linked via media coverage to primarily White suburban and rural communities, while heroin, methamphetamines, and cocaine are linked to racial minorities in urban spaces (Netherland & Hansen, 2016). Given the stark differences between the way these drug types are perceived, it is likely that perceived stigma towards these drug types would have varied impacts on drug-related knowledge.

Our survey questions assessing stigma are based on the “awareness” portion of the brief opioid stigma scale (i.e., “My community thinks that [PWUD stereotype]”) which measures stigma at a step removed from “agreement” (i.e., “I think that [PWUD stereotype]”) (Yang, 2019). Further study should investigate the mechanism driving the opposite effects of perceived stigma towards people who use opioids and people who use heroin, methamphetamines, and cocaine, and whether these effects extend to more internalized measures of stigma.

Our study also finds that those who are familiar with SSPs are 80.9% more likely to be familiar with naloxone, 164.9% more likely to know where to access it, and 299.7% more likely to know how to use it. This result corresponds with evidence that using SSPs is correlated with increased naloxone possession (Jones, 2021; Lipira, 2021; Reed, 2019). Our result is notable because SSPs are banned in Nebraska, and we are unaware of any unsanctioned distribution efforts (Nebraska Revised Statute 28–442, 2017). Any benefit emerging from familiarity with SSPs is not due to syringe services provided within Nebraska. Legalizing SSPs, which almost always facilitate naloxone distribution programs (Lambdin et al., 2020), in Nebraska may further increase the positive association between familiarity with SSPs and likelihood of naloxone familiarity, access, or competency.

Higher rates of religious attendance are associated with lower likelihood of naloxone familiarity, but have no effect on naloxone access and competency among those with familiarity. This finding aligns with previous research indicating that areas with high religious adherence often lack substance use disorder treatment programs (Woodruff & Frakt, 2020). These results suggest that campaigns aiming to promote naloxone familiarity could benefit from developing tailored messaging and outreach strategies for religious communities. This tactic is not unprecedented: places of worship have successfully implemented programs aimed at preventing HIV and substance abuse (Francis & Liverpool, 2009; Johnson et al., 2000) and the Substance Abuse and Mental Health Services Administration offers support for faith-based organizations implementing overdose prevention programs (Woodruff & Frakt, 2020).

We notably do not find any significant effects linked to gender or race, in contrast with previous studies. Being female compared to being male was positively associated with naloxone familiarity (Schlosser, 2022) and being White compared to being Black or Hispanic was positively associated with the likelihood of having naloxone training (Khan et al., 2023).

Our Nebraska-wide finding of no significant relationship between lifetime illicit opioid use, being close to a past-year overdose, or being close to a person who uses illicit opioids and likelihood of naloxone knowledge is especially important. People in these groups may be more likely to be in situations where naloxone use is necessary, but according to our results are not more likely to have naloxone familiarity, access, or competency. In our subpopulation analysis, we find region-dependent variation in these effects. In Omaha and Lincoln, there is no association between naloxone knowledge and being in groups where naloxone use is more likely to be necessary. While we found an association between these groups and naloxone knowledge in regions outside Omaha and Lincoln, promoting naloxone knowledge among rural individuals close to opioid use remains important. In rural areas, non-professional possession of naloxone is critical because of slower emergency response times, lower rate of naloxone administration by first responders, and fewer bystanders (Cabral, 2018; Faul et al., 2015).

## Limitations

Our findings have several limitations. First, our measurements of naloxone knowledge are self-reported in a yes/no format rather than asking skill questions (e.g., identifying correct ways to administer naloxone or a location they could obtain naloxone). If respondents exaggerated their knowledge of naloxone, our estimates of overall naloxone knowledge in Nebraska would be biased upwards compared to a skill-focused measure of knowledge used in prior studies (Hohmann, 2022). Using binary variables to measure naloxone knowledge does not capture nuance such as how many sources of naloxone a respondent could identify or how quickly they could administer naloxone in an emergency. Future research in this area will be needed to build on the present findings.

Finally, the Nebraska Annual Social Indicators Survey relies solely on address-based sampling, which has a relatively high response rate and extensive coverage (Link et al., 2008). Despite these benefits, address-based sampling does not reach the unhoused population and can undercount certain demographics including rural areas (Dohrmann et al., 2006; O’Muircheartaigh et al., 2007). To ensure higher levels of participation in rural areas of the state, a stratified sample design was used to include more participants from outside urban areas. The Nebraska age of majority prevents those under 19 years old from completing the survey, excluding 18-year-olds from the sample. This age restriction is a unique feature of Nebraska-specific research. Sample size restrictions also prevented highly specified subpopulation regressions with our model. These restrictions should remain in mind when interpreting our findings.

## Conclusion

Our address-based sample of Nebraska residents shows significant gaps in the naloxone treatment cascade between naloxone familiarity and both access and competency, suggesting the need for increased efforts to increase these factors. People who have used illicit opioids in their lifetime, were close to a past year overdose, and were close to a person who uses illicit opioids, as well as those with higher rates of religious service attendance are all demographic groups that may have less likelihood of naloxone knowledge. Measures that focus on these groups have the most room to increase naloxone knowledge. We found that familiarity with syringe service programs was positively associated with naloxone knowledge, and the Nebraska legislature should consider legalizing syringe service programs to further increase this association. Finally, future work should examine the varied impact of perceived substance-specific stigma on naloxone knowledge. Understanding nuances in stigma towards people who use drugs could lead to more efficient and inclusive methods of naloxone distribution in the future.

## Figures and Tables

**Table 1:** Descriptive Statistics

Measure (n = 1,455, m = 50)	Mean/Proportion	Standard Error of Estimate	95% Confidence Interval of Estimate	
Mean Age	49.39	0.65	48.11	50.65
Gender				
Male	49.81	2.02	45.86	53.77
Female	50.19	2.02	46.23	54.14
Race/Ethnicity				
White non-Hispanic	90.81	1.20	88.45	93.16
Non-White/Hispanic	9.19	1.20	6.84	11.55
Education				
No College	16.59	1.36	13.92	19.26
Technical Degree/Some College	29.56	1.8	26.03	33.1
B.A./Terminal Degree	53.84	1.98	49.97	57.72
Employment				
Unemployed	24.2	1.48	21.30	27.11
Employed	75.8	1.48	72.89	78.7
Annual Income				
$0 - $30,000	11.12	1.06	9.04	13.2
$30,001 - $100,000	47.91	2.03	43.92	51.9
$100,001 +	40.97	2.03	36.99	44.96
Partner Status				
No Partner	24.82	1.60	21.68	27.95
Married/With Partner	75.18	1.60	72.05	78.32
Mean Children in Household	0.79	0.05	0.68	0.89
Political Orientation				
Liberal	22.85	1.78	19.36	26.35
Moderate	36.51	2.02	32.54	40.47
Conservative	40.64	1.94	36.83	44.45
Religious Denomination				
Protestant	42.69	1.97	38.83	46.56
Catholic	22.87	1.63	19.68	26.07
No Affiliation	22.8	1.82	19.23	26.37
Other	11.63	1.31	9.06	14.21
Religious Attendance	2.98	0.11	2.76	3.19
Core-Based Statistical Area				
Town or City	83.14	1.21	80.77	85.51
Open Country; not Farm	8.75	0.96	6.86	10.64
Farm	8.11	0.88	6.37	9.84
Core-Based Statistical Areas				
Metropolitan	68.4	1.23	65.99	70.81
Micropolitan	13.69	0.95	11.83	15.54
Counties Outside	17.91	1.08	15.79	20.03
Close to Past-year Overdose (y/n)	5.87	0.96	3.98	7.76
Lifetime Opioid or Heroin Use (y/n)	6.56	1.19	4.23	8.89
Close to Opioid or Heroin Use (y/n)	6.30	1.08	4.18	8.41
Access to Opioids or Heroin (y/n)	16.18	1.55	13.13	19.23
Community Opioid Stigma	3.49	0.03	3.42	3.55
Community Heroin, Methamphetamine, and Cocaine Stigma	3.70	0.03	3.64	3.76
Familiarity with SSPs (y/n)	30.84	1.96	27.00	34.69
Familiarity with Naloxone (y/n)	75.60	1.64	72.38	78.81
Access to Naloxone (y/n)	18.59	1.56	15.52	21.66
Naloxone Competency (y/n)	17.61	1.56	14.54	20.67
Access to Naloxone among those Familiar (n = 1,097)	24.46	1.95	20.62	28.29
Naloxone Competency among those Familiar (n = 1,097)	23.02	1.96	19.19	26.86

**Table 2:** Logistic regression models predicting knowledge of naloxone (m = 50)

Measure	Naloxone Familiarity (n = 1,455)				Naloxone Access Among those with Familiarity (n = 1,097)				Naloxone Competency Among those with Familiarity (n = 1,097)			
	OR	*p* value	95% Conf. Interval		OR	*p* value	95% Conf. Interval		OR	*p* value	95% Conf. Interval	
Age	1.000	1.000	0.986	1.014	1.015	0.112	0.997	1.034	0.998	0.870	0.979	1.018
Gender												
Male	ref.				ref.				ref.			
Female	1.297	0.153	0.908	1.853	1.233	0.374	0.777	1.957	1.498	0.102	0.923	2.430
Race/Ethnicity												
White non-Hispanic	ref.				ref.				ref.			
Non-White/Hispanic	0.937	0.846	0.483	1.816	1.204	0.674	0.508	2.852	1.259	0.618	0.509	3.115
Education												
No College	ref.				ref.				ref.			
Technical Degree/Some College	*1.748*	*0.027*	*1.066*	*2.864*	1.686	0.162	0.810	3.512	1.335	0.472	0.607	2.937
B.A./Terminal Degree	1.279	0.340	0.771	2.122	1.694	0.158	0.815	3.519	1.300	0.493	0.614	2.756
Employment												
Unemployed	ref.				ref.				ref.			
Employed	0.829	0.403	0.535	1.286	*1.957*	*0.016*	*1.136*	*3.370*	1.652	0.132	0.859	3.175
Annual Income												
$0 - $30,000	ref.				ref.				ref.			
$30,001 - $100,000	1.391	0.226	0.815	2.373	0.959	0.920	0.423	2.172	0.837	0.673	0.365	1.917
$100,001 +	*2.112*	*0.032*	*1.067*	*4.181*	0.936	0.886	0.375	2.334	0.781	0.596	0.313	1.951
Partner Status												
No Partner	ref.				ref.				ref.			
Married/With Partner	1.189	0.417	0.783	1.804	1.345	0.280	0.785	2.302	1.372	0.253	0.798	2.362
Political Orientation												
Liberal	ref.				ref.				ref.			
Moderate	0.959	0.892	0.522	1.760	0.893	0.718	0.484	1.648	*2.209*	*0.024*	*1.109*	*4.400*
Conservative	0.613	0.116	0.333	1.128	0.635	0.165	0.334	1.205	1.374	0.406	0.649	2.906
Religious Denomination												
Protestant	ref.				ref.				ref.			
Catholic	0.969	0.890	0.623	1.507	1.225	0.495	0.684	2.195	1.921	0.039	1.034	3.570
No Affiliation	0.753	0.373	0.404	1.406	0.953	0.896	0.462	1.965	1.075	0.859	0.482	2.402
Other	0.850	0.609	0.455	1.587	1.207	0.614	0.582	2.503	1.091	0.839	0.470	2.530
Religious Attendance	*0.875*	*0.004*	*0.798*	*0.959*	1.024	0.683	0.914	1.147	1.004	0.953	0.877	1.149
Residence												
City or Town	ref.				ref.				ref.			
Open Country; not a Farm	1.058	0.845	0.599	1.870	1.017	0.963	0.508	2.034	1.128	0.765	0.512	2.486
Farm	0.769	0.390	0.422	1.401	1.259	0.595	0.539	2.940	0.751	0.606	0.252	2.233
Core-Based Statistical Areas												
Metropolitan	ref.				ref.				ref.			
Micropolitan	0.884	0.590	0.565	1.384	1.279	0.366	0.750	2.181	0.916	0.755	0.527	1.592
Counties Outside	0.762	0.222	0.493	1.179	1.190	0.583	0.639	2.214	0.751	0.360	0.407	1.387
Close to Past-year Overdose (y/n)	0.675	0.393	0.275	1.661	2.239	0.079	0.912	5.498	2.478	0.053	0.990	6.202
Lifetime Opioid or Heroin Use (y/n)	0.999	0.999	0.341	2.923	0.533	0.191	0.208	1.369	0.899	0.828	0.345	2.347
Close to Opioid or Heroin Use (y/n)	0.902	0.856	0.294	2.768	0.664	0.450	0.229	1.926	0.496	0.240	0.154	1.598
Access to Opioids or Heroin (y/n)	1.194	0.523	0.692	2.061	*2.274*	*0.006*	*1.268*	*4.079*	1.640	0.119	0.881	3.053
Community Opioid Stigma	0.962	0.822	0.683	1.354	*0.464*	*0.001*	*0.301*	*0.715*	*0.611*	*0.036*	*0.385*	*0.969*
Community Heroin, Methamphetamine, and Cocaine Stigma	1.189	0.343	0.831	1.701	*2.154*	*0.002*	*1.331*	*3.486*	1.346	0.235	0.824	2.196
Familiarity with SSPs (y/n)	*1.809*	*0.015*	*1.125*	*2.910*	*2.649*	*0.000*	*1.652*	*4.247*	*3.997*	*0.000*	*2.414*	*6.617*
Constant	1.218	0.797	0.270	5.487	*0.019*	*0.000*	*0.002*	*0.154*	*0.071*	*0.019*	*0.008*	*0.647*

**Table 3a:** Logistic regression subgroup models predicting knowledge of naloxone in Omaha and Lincoln (m = 50)

Measure	Naloxone Familiarity (n = 698)				Naloxone Access Among those with Familiarity (n = 565)				Naloxone Competency Among those with Familiarity (n = 565)			
	OR	*p* value	95% Conf. Interval		OR	*p* value	95% Conf. Interval		OR	*p* value	95% Conf. Interval	
Age	0.978	0.110	0.951	1.005	1.013	0.467	0.978	1.049	0.981	0.267	0.948	1.015
Gender												
Male	ref.				ref.				ref.			
Female	1.450	0.354	0.659	3.191	1.083	0.840	0.498	2.356	1.796	0.133	0.836	3.858
Race/Ethnicity												
White non-Hispanic	ref.				ref.				ref.			
Non-White/Hispanic	1.733	0.356	0.538	5.586	1.305	0.687	0.356	4.783	1.241	0.757	0.316	4.876
Education												
No College	ref.				ref.				ref.			
Technical Degree/Some College	2.060	0.216	0.655	6.479	4.454	0.176	0.510	38.873	6.363	0.159	0.482	84.032
B.A./Terminal Degree	0.820	0.718	0.279	2.410	3.437	0.255	0.409	28.885	7.118	0.131	0.555	91.235
Employment												
Unemployed	ref.				ref.				ref.			
Employed	*0.326*	*0.016*	*0.131*	*0.809*	*4.505*	*0.006*	*1.546*	*13.124*	1.837	0.297	0.584	5.776
Annual Income												
$0 - $30,000	ref.				ref.				ref.			
$30,001 - $100,000	2.544	0.133	0.751	8.614	0.351	0.155	0.083	1.489	0.618	0.520	0.142	2.685
$100,001 +	*6.267*	*0.012*	*1.506*	*26.085*	0.237	0.075	0.048	1.159	0.509	0.400	0.106	2.458
Partner Status												
No Partner	ref.				ref.				ref.			
Married/With Partner	1.070	0.872	0.469	2.442	1.385	0.485	0.554	3.465	1.273	0.601	0.514	3.148
Political Orientation												
Liberal	ref.				ref.				ref.			
Moderate	0.695	0.526	0.225	2.149	0.759	0.560	0.299	1.925	*3.047*	*0.036*	*1.073*	*8.653*
Conservative	0.344	0.076	0.106	1.120	0.506	0.271	0.150	1.705	1.461	0.567	0.397	5.371
Religious Denomination												
Protestant	ref.				ref.				ref.			
Catholic	0.431	0.195	0.120	1.545	1.117	0.864	0.314	3.976	1.217	0.777	0.310	4.781
No Affiliation	0.620	0.284	0.258	1.489	1.292	0.626	0.460	3.627	2.631	0.054	0.984	7.034
Other	0.619	0.419	0.193	1.986	1.069	0.917	0.302	3.788	1.051	0.941	0.283	3.905
Religious Attendance	*0.814*	*0.048*	*0.664*	*0.998*	1.038	0.735	0.834	1.292	1.042	0.735	0.821	1.323
Close to Past-year Overdose (y/n)	1.163	0.921	0.058	23.376	0.725	0.709	0.133	3.948	1.550	0.586	0.318	7.550
Lifetime Opioid or Heroin Use (y/n)	0.236	0.155	0.032	1.733	0.558	0.524	0.092	3.369	1.273	0.785	0.223	7.282
Close to Opioid or Heroin Use (y/n)	0.569	0.590	0.073	4.449	0.837	0.855	0.125	5.622	0.742	0.778	0.093	5.943
Access to Opioids or Heroin (y/n)	2.647	0.061	0.956	7.325	1.440	0.490	0.509	4.072	1.102	0.865	0.358	3.394
Community Opioid Stigma	1.249	0.523	0.631	2.471	*0.371*	*0.009*	*0.177*	*0.780*	0.736	0.420	0.349	1.553
Community Heroin, Methamphetamine, and Cocaine Stigma	1.017	0.961	0.511	2.025	*3.252*	*0.007*	*1.377*	*7.679*	0.910	0.811	0.419	1.974
Familiarity with SSPs (y/n)	2.445	0.056	0.979	6.109	1.777	0.174	0.775	4.075	*3.315*	*0.005*	*1.445*	*7.604*
Constant	11.463	0.113	0.558	235.655	0.010	0.010	0.000	0.331	0.073	0.135	0.002	2.265

**Table 3b:** Logistic regression subgroup models predicting knowledge of naloxone in regions outside Omaha and Lincoln (m = 50)

Measure	Naloxone Familiarity (n = 757)				Naloxone Access Among those with Familiarity (n = 529)				Naloxone Competency Among those with Familiarity (n = 529)			
	OR	*p* value	95% Conf. Interval		OR	*p* value	95% Conf. Interval		OR	*p* value	95% Conf. Interval	
Age	1.013	0.066	0.999	1.028	*1.022*	*0.044*	*1.001*	*1.044*	1.013	0.236	0.991	1.035
Gender												
Male	ref.				ref.				ref.			
Female	1.182	0.411	0.793	1.760	1.298	0.345	0.755	2.229	1.255	0.434	0.710	2.218
Race/Ethnicity												
White non-Hispanic	ref.				ref.				ref.			
Non-White/Hispanic	0.642	0.222	0.315	1.308	0.908	0.876	0.269	3.066	0.875	0.842	0.234	3.267
Education												
No College	ref.				ref.				ref.			
Technical Degree/Some College	1.644	0.061	0.977	2.765	0.929	0.850	0.431	2.001	0.669	0.315	0.305	1.467
B.A./Terminal Degree	1.642	0.069	0.962	2.802	1.084	0.837	0.503	2.335	0.493	0.092	0.216	1.122
Employment												
Unemployed	ref.				ref.				ref.			
Employed	1.267	0.317	0.797	2.012	1.249	0.515	0.639	2.441	1.624	0.210	0.761	3.465
Annual Income												
$0 - $30,000	ref.				ref.				ref.			
$30,001 - $100,000	1.142	0.637	0.658	1.979	2.079	0.059	0.971	4.452	0.909	0.846	0.344	2.399
$100,001 +	1.425	0.313	0.716	2.835	*2.799*	*0.021*	*1.171*	*6.691*	1.076	0.890	0.377	3.075
Partner Status												
No Partner	ref.				ref.				ref.			
Married/With Partner	1.245	0.319	0.809	1.916	1.408	0.272	0.765	2.590	1.920	0.075	0.935	3.943
Political Orientation												
Liberal	ref.				ref.				ref.			
Moderate	1.301	0.408	0.697	2.429	1.022	0.957	0.463	2.254	1.493	0.368	0.622	3.579
Conservative	0.898	0.734	0.484	1.668	0.853	0.676	0.403	1.804	1.042	0.928	0.429	2.528
Religious Denomination												
Protestant	ref.				ref.				ref.			
Catholic	0.895	0.748	0.456	1.758	0.894	0.793	0.386	2.071	0.864	0.769	0.327	2.287
No Affiliation	1.120	0.646	0.690	1.820	1.159	0.669	0.588	2.284	1.506	0.305	0.688	3.296
Other	1.017	0.962	0.509	2.035	1.495	0.352	0.640	3.491	1.164	0.776	0.408	3.322
Religious Attendance	*0.896*	*0.024*	*0.814*	*0.986*	1.021	0.741	0.902	1.155	0.949	0.574	0.792	1.138
Close to Past-year Overdose (y/n)	0.518	0.092	0.241	1.114	*4.806*	*0.001*	*1.836*	*12.579*	*3.465*	*0.009*	*1.371*	*8.760*
Lifetime Opioid or Heroin Use (y/n)	2.044	0.149	0.774	5.396	0.843	0.767	0.273	2.607	0.437	0.097	0.165	1.163
Close to Opioid or Heroin Use (y/n)	1.428	0.450	0.566	3.602	0.575	0.297	0.203	1.627	0.742	0.565	0.268	2.053
Access to Opioids or Heroin (y/n)	1.035	0.907	0.576	1.861	*3.234*	*0.001*	*1.606*	*6.516*	*2.166*	*0.036*	*1.054*	*4.453*
Community Opioid Stigma	0.832	0.356	0.562	1.230	0.613	0.053	0.374	1.007	0.607	0.060	0.361	1.022
Community Heroin, Methamphetamine, and Cocaine Stigma	1.300	0.226	0.850	1.989	1.383	0.239	0.805	2.375	1.595	0.134	0.865	2.939
Familiarity with SSPs (y/n)	1.471	0.148	0.872	2.482	*4.367*	*<0.001*	*2.573*	*7.411*	*6.215*	*<0.001*	*3.408*	*11.335*
Constant	0.403	0.367	0.056	2.913	0.018	0.005	0.001	0.297	0.029	0.013	0.002	0.477

## Data Availability

The data analyzed during this study—the 2022 Nebraska Annual Social Indicators Survey—will be publicly available upon request to the Bureau of Sociological Research of the University of Nebraska-Lincoln after December 1, 2023.

## Abbreviations

SSPs: Syringe service programs
PWUD: People who use drugs
